# The Effects of Virtual Reality–Based Reminiscence Therapies for Older Adults With Cognitive Impairment: Systematic Review

**DOI:** 10.2196/53348

**Published:** 2024-11-12

**Authors:** Qian Mao, Zhen Zhao, Lisha Yu, Yang Zhao, Hailiang Wang

**Affiliations:** 1 School of Design The Hong Kong Polytechnic University Hong Kong China; 2 Division of Artificial Intelligence Lingnan University Hong Kong China; 3 School of Public Health (Shenzhen) Sun Yat-sen University Shenzhen China

**Keywords:** virtual reality, reminiscence therapy, cognitive impairment, older adults, mobile phone

## Abstract

**Background:**

Reminiscence therapy (RT) is a commonly used nonpharmaceutical treatment for cognitive impairment. Artifacts or conversations are used in RT to recall individuals’ memories and past experiences. Virtual reality (VR) has increasingly been used as an assistive technology during RT. However, the effects of VR-based RT (VR-RT) methods remain unclear, and insights into the related benefits and challenges are urgently needed.

**Objective:**

The study aims to systematically review the effects of VR-RTs for older adults with cognitive impairment.

**Methods:**

Seven databases (MEDLINE, Academic Search Premier, CINAHL, Web of Science, PubMed, the Cochrane Central Register of Controlled Trials, and ScienceDirect) were searched to identify relevant articles published from inception to August 10, 2023. Peer-reviewed publications that assessed the effect of VR-RTs (ie, using virtual clues to evoke participants’ memories or past experiences) on cognitive-related outcomes were included. Two independent researchers conducted the literature search, review, and data extraction processes. A narrative synthesis approach was used to analyze the extracted data.

**Results:**

Of the 537 identified articles, 22 were ultimately included in the data analysis. The results revealed that VR-RTs could maintain cognitive status (4/4, 100%) and reduce anxiety (2/2, 100%) in older adults with cognitive impairment. Nevertheless, one study found a cognitive improvement after VR-RTs, whereas cognitive degradation was observed at a 3- to 6-month follow-up measure. Around 88% (7/8) of the included studies indicated that VR-RTs improved memory; however, the evidence regarding the beneficial effects of VR-RTs was limited in improving quality of life (1/4, 25%) and reducing apathy (0/2, 0%) and depression (1/3, 33%). The results indicated that VR-RTs are safe, engaging, acceptable, and satisfying for older adults with cognitive impairment. In VR scenarios, personalized stimulus materials related to the users’ youth experiences were more effective for treating cognitive impairment than other stimulus materials.

**Conclusions:**

The results of this systematic review demonstrate the potential benefits of VR-RT for older adults with cognitive impairment, especially in improving emotion and memory and maintaining cognitive status. VR-RT is also safe and enjoyable for older adults. However, due to the trial heterogeneity of included studies, we can only provide qualitative results instead of performing meta-analysis to quantify the effect size of VR-RTs. Thus, more randomized controlled trials are required to examine the designs and effects of VR-RTs for groups of older adults with specific needs.

## Introduction

### Cognitive Impairment

The World Health Organization predicts that by 2050, the proportion of older adults worldwide will double and thus account for approximately a quarter of the global population [1]. Many older adults experience cognitive impairment, such as losing memory or other cognitive abilities (eg, language, visual, and spatial perception), as they age [2]. Approximately 5% to 10% of older adults diagnosed with mild cognitive impairment (MCI) develop dementia each year [3], a condition that negatively affects their neuropsychiatric health, as exemplified by the manifestation of anxiety, depression, social isolation, and sleep disorders [4]. Dementia also severely affects the independence of older adults and their ability to function in daily life, thus reducing their quality of life [5]. Dementia further places substantial socioeconomic and care burdens on the family members of affected individuals, health care systems, and even societies. The total global cost associated with dementia, which affects 55.2 million people worldwide, is now estimated to be US $800 billion per year and is predicted to increase to US $2 trillion by 2030 [6]. Approximately 50% of these costs can be attributed to informal care, who were found to deliver an average of 5 hours of care and supervision per day [7]. Therefore, it is becoming increasingly important to address cognitive impairment and defer its progression through timely diagnosis and the provision of effective interventions at an early stage.

Traditional interventions for cognitive impairment include both pharmacological and nonpharmacological therapies. Pharmacological therapies (eg, atypical antipsychotic medications) are major interventions for older adults with cognitive impairment and aim to alleviate symptoms rather than the underlying disease processes [8]. Research efforts have been expanded to nonpharmacological therapies, which can slow functional decline, improve apathy, reduce disruptive behaviors, and slow the progression from cognitive impairment to dementia [9]. Among these, reminiscence therapy (RT) involves the organized discussion and recall of past life experiences guided by props such as videos, pictures, locations, and objects [10]. This approach may improve memory and depressive symptoms in older adults with cognitive impairment [11]. For example, some studies have shown the beneficial effects of personal reminiscing namely, mental stimulation, social interaction, and emotional connections, which can improve older adults’ mood, self-esteem, and overall quality of life [12,13]. Older adults can easily perform such RT interventions, and they can be administered by therapists or researchers in various settings [14]. However, traditional methods of RT have some drawbacks that have limited its widespread application. For example, RT requires face-to-face interaction between the therapist and participant during treatment sessions, which may be limited by the available physical space [15]. Another potential limitation is the availability of the interactive resources (eg, personalized props and scenes) needed to offer a highly immersive environment that can evoke emotional responses during the interactions [16]. Thus, some researchers are attempting to support RT interventions by incorporating virtual reality (VR) technologies.

### VR-Based RTs

VR refers to an immersive and interactive computer-generated environment that simulates a real or imagined experience [17]. A VR user wears a head-mounted display that provides auditory, visual, and tactile feedback to enhance their sense of presence in the virtual world [18]. In the context of cognitive impairment, VR technology has emerged as a promising intervention and support tool that can enhance cognitive abilities and promote the engagement of older adults [19]. Recent work has underlined the benefits of VR technology in interventions for cognitive impairment. For example, Dockx et al [20] indicated that VR technology’s immersive and interactive nature enhanced users’ engagement, enjoyment, and therapy adherence. Tarnanas et al [21] emphasized that VR technology offers a safe and controlled environment for practicing cognitive skills as it eliminates the risks associated with real-world activities. VR technology also gives therapists much control over the therapy experience. Liu et al [22] showed that VR technology has surpassed the traditional paper-and-pencil assessment paradigm and can capture users’ behavioral data, thus allowing the accurate assessment and identification of people with cognitive impairment. Through controlled experiments, Liao et al [23] found that VR technology significantly improved executive function in older adults with cognitive impairment (*P*=.03). In summary, the controlled environment, tailored training, immediate feedback, and engagement provided through VR interventions contribute to the cognitive-related benefits among older adults.

Given these effects of VR technology in the context of cognitive impairment interventions, many researchers have combined VR technology with RT, and their studies have provided new evidence for the effectiveness of VR-based RTs (VR-RTs). VR-RTs can improve memory recall by providing cues and prompts (eg, movies, videos, images, or 3D-rendered scenes) that trigger specific memories in the user. Its immersive nature can enhance the retrieval of detailed and vivid memories, thus stimulating cognitive processes and strengthening neural connections [24]. For example, Xu and Wang [25] used a VR system to restore life scenes in China in the 1970s. They demonstrated that this VR-based RT (VR-RT) intervention was highly effective and acceptable among older adults with cognitive impairment. In addition, Saredakis et al [26] found that VR-RT could reduce symptoms of isolation and apathy among older adults with cognitive impairment. Hence, VR-RTs have great potential to alleviate memory loss and promote communication, cognition, and positive mood.

Prior reviews have focused predominantly on either VR technology or RT as interventions for cognitive impairment and synthesized the existing VR technology landscape or the impacts of RT interventions on individuals with cognitive impairment [22,27,28]. To the best of our knowledge, only the study by Reisinho et al [29] has reviewed the applications, benefits, and limitations of VR-RTs for the cognitive rehabilitation of people with cognitive impairment or dementia. However, that review paid little attention to the VR-RT stimulus materials and construction methods, synthesis of the effectiveness of VR-RTs for older adults with cognitive impairment, or user satisfaction. Attention to such aspects is required to optimize the design and implementation of VR-based cognitive rehabilitation programs, enhance users’ engagement, and improve users’ cognitive functioning.

We conducted a systematic review to explore and synthesize the evidence of the effects and applicability of VR-RTs among older adults with cognitive impairment. Specifically, this review summarizes the materials, construction methods, and intervention designs of existing VR-RTs. Then, we review and analyze the effectiveness of VR-RTs in improving the quality of life and cognitive-related functions of older adults with cognitive impairment. Finally, we discuss the user satisfaction and side effects of VR-RTs. The findings of this review have led us to provide valuable recommendations for researchers, designers, and caregivers who use VR-RTs to care for older adults with cognitive impairment.

## Methods

### Search Strategy

Seven electronic databases namely, MEDLINE, Academic Search Premier, CINAHL, Web of Science, PubMed, the Cochrane Central Register of Controlled Trials, and ScienceDirect, were searched for relevant articles up to August 10, 2023.

We used the following search terms and search strategy for potential studies related to VR-based RTs among older adults with cognitive impairments: (virtual reality OR virtual OR VR OR virtual simulation) AND (reminiscence OR narrative OR life review OR life story book) AND (dementia OR cognitive impairment OR MCI OR Alzheimer’s disease OR memory disorder OR cognitive decline OR cognitive dysfunction OR memory impairment).

### Study Selection

Studies were screened on the basis of the inclusion and exclusion criteria, as shown in Textbox 1. A 3-step screening process was implemented to ensure the comprehensiveness and validity of our systematic review. First, 2 authors (QM and ZZ) independently identified relevant articles during the initial review by screening the titles and abstracts. Second, the full texts of the relevant articles were reviewed to determine their eligibility according to the predefined inclusion and exclusion criteria. Third, the reference lists of the included articles were manually examined to identify any missed studies, and the Google Scholar database was manually searched using search terms and search strategy to identify additional potentially relevant studies. The authors independently assessed the articles in all review steps. Any discrepancies regarding study inclusion were discussed with a third author (HW) until a final decision was reached.

**The inclusion and exclusion criteria for screening studies related to virtual reality–based reminiscence therapies (RT) among older adults with cognitive impairments.**

**Inclusion criteria**

- Article type: used RT, a psychosocial intervention using prompts to evoke participants’ memories or discussing participants’ past events and experiences, and virtual reality (VR) technology; evaluated the effects of VR-based RTs on individuals with cognitive impairment; published in a peer-reviewed journal or conference proceedings.
- Language: written in English.

**Exclusion criteria**

- Article type: did not use RT and VR for cognitive impairment intervention; did not evaluate the effects of VR-RTs; books, review articles, and commentary letters, as well as unpublished papers.
- Language: papers not written in English.

### Data Extraction

Textbox 2 presents the types of information and data extracted independently by 2 authors (QM and ZZ). Specifically, the beneficial effects of VR-RTs on the quality of life and cognitive-related functions of people with cognitive impairment were summarized. “Quality of life” refers to the overall mental and physical health in daily life. Cognitive-related functions refer to neuropsychiatric issues due to cognitive impairment, such as anxiety and declines in memory and cognitive status. The analysis of user satisfaction was based on side effects and user acceptability, immersion, and engagement. “Side effects” refer to various forms of physical discomfort triggered by VR-RTs, such as eye strain, vertigo, or nausea. “User acceptability” mainly refers to users’ attitudes toward the interventions and reveals the level of acceptance of VR-RTs. Immersion indicates the degree of immersiveness of the VR environment, and engagement indicates the level of individuals’ active and meaningful participation in an intervention.

**The extracted information on study design, participants, interventions, and outcomes.**

**Study**

- First author
- Year of publication
- Study region
- Study aim
- Study design

**Participants**

- Sample size
- Average age
- Sex distribution
- Health status

**Virtual reality (VR) technology**

- Device
- Locations of device
- System type
- Construction approach

**VR-based reminiscence therapy**

- Contents of the VR-based reminiscence therapy
- Intervention frequency, duration per session, and intervention duration

**Outcome measure**

- Quality of life
- Cognitive-related functions
- User satisfaction

### Data Synthesis

We qualitatively synthesized the included articles to analyze the effects and applicability of VR-RTs among older adults with cognitive impairment. Specifically, we identified the studies and participants’ characteristics. We then summarized and analyzed the VR technologies and intervention designs of the VR-RTs according to the extracted data.

We classify, herein, VR technologies into nonimmersive, semi-immersive, or immersive VR technologies [30]. Specifically, nonimmersive VR technologies generally use small platform screens, such as tablets, computers, and televisions, to produce a VR environment with low levels of immersion and interaction. Semi-immersive VR technologies, ranked between nonimmersive and immersive VR, allow users to engage in a partially virtual environment with certain degrees of immersion and interactivity using large-screen projectors and panoramic monitors. In immersive VR, the virtual environment completely replaces the physical world through platforms that provide full views and sensory feedback, such as head-mounted displays and cave automatic virtual environment.

The effectiveness of VR-RTs in improving quality of life and cognitive-related functions was further analyzed according to the differences in the values of the outcomes from before to after the intervention. On the basis of the above-mentioned synthesis, we critically discuss the benefits and challenges of applying VR technologies during RT for older adults with cognitive impairment.

### Risk of Bias Assessment

The Cochrane Risk of Bias in Non-Randomized Studies of Interventions tool was used independently by 2 authors (QM and ZZ) to assess the risk of bias in the included studies. The assessment indices of the tool consist of 7 domains, including confounding, selection of participants in the study, classification of interventions, deviations from intended interventions, missing data, measurement of outcomes, and selection of the reported result. Each domain was classified into low risk, moderate risk, serious risk, or critical risk on the basis of the responses to the items, and an overall assessment was calculated based on the 7 domains [31].

## Results

### Study Identification

**Figure 1:**
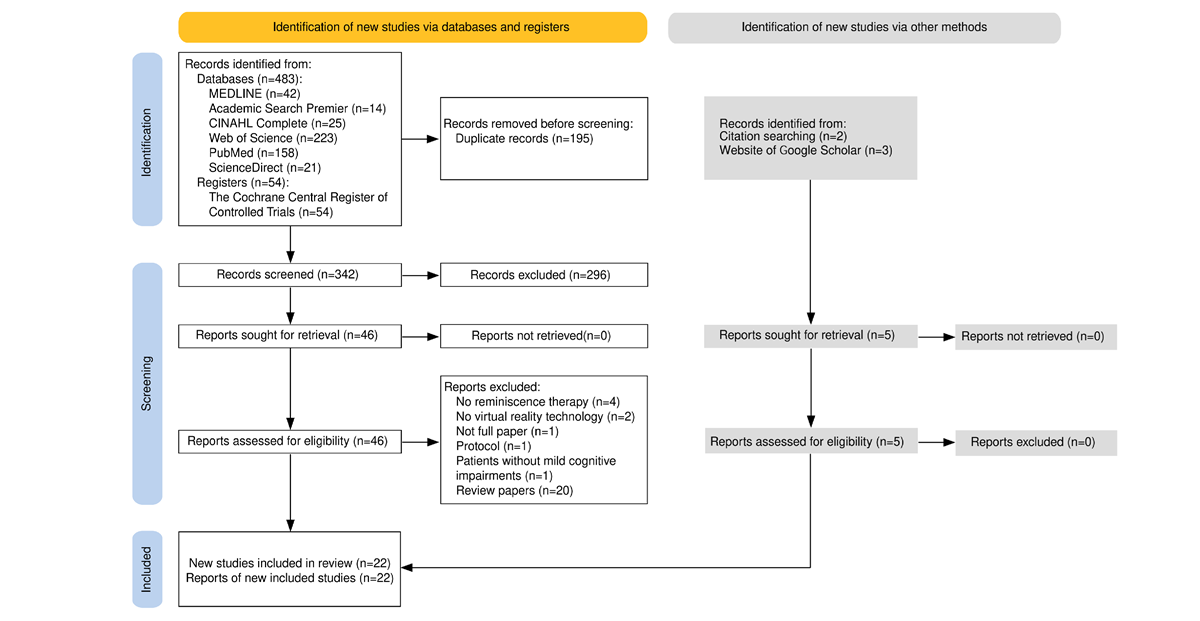
PRISMA (Preferred Reporting Items for Systematic Reviews and Meta-Analyses) diagram of the literature search and review process for potential articles on virtual reality-based reminiscence therapies among older adults with cognitive impairments.

We identified 537 articles in our initial search, of which, 195 were excluded as duplicates. Two authors (QM and ZZ) screened the titles and abstracts of the remaining 342 articles to identify potentially relevant studies. We reviewed the full texts of 46 potentially relevant articles, and 17 articles met our inclusion criteria. We further identified 5 potentially eligible articles by manually searching the reference lists of the included articles and the Google Scholar database for missed studies. Finally, 22 articles were included in the systematic review. Figure 1 presents the literature search and review process.

### Study Characteristics

The 22 included studies comprised 1 (5%) case study [32], 1 (5%) randomized controlled trial (RCT) study [24], 7 (32%) pilot studies [33-39], 6 (27%) feasibility studies [25,26,40-43], and 7 (32%) prototype studies [18,44-49]. Most of the included studies were conducted in Australia (3/22, 14%) [26,33,35], France (3/22, 14%) [41,42,46], Japan (3/22, 14%) [24,32,39], or Taiwan (3/22, 14%) [37,44,45]. Half of the studies were published in the year 2020 [25,26,35,36,43,47] or 2021 [24,32,33,39,40].

The sample sizes in the included studies ranged from 2 to 63 participants. The mean age of the participants ranged from 63.40 to 89.80 years. Female accounted for 38% (5/13) to 89% (39/44) of the participants in the included studies. More information on the 22 included articles can be found in Multimedia Appendix 1. The detailed information on the PRISMA checklist can be found in Multimedia Appendix 2.

### VR Technology

Typically, in the included studies on nonimmersive VR, a computer was used to present scenes [24,34,38,43,45,47,49]. The participants used a Kinect (Microsoft Corporation) [45], mouse and keyboard [34,38,43,47,49], or tactile screen [24] to interact with the VR environment. In semi-immersive VR, BARCO iSpace (Barco NV) or a large projector was used to show the VR environment [41,42,46,48], and users used an ART wireless finger-tracking [41,46], mouse [42], or a Kinect [48] to interact with the environment. Immersive VR was the most popular technology, as it was used in about 60% (13/22) of the included studies [18,25,26,32,33,35-37,39,40,44,47,49]. Oculus (Meta Platforms) (8/22, 36%) and VIVE (HTC Corporation) headsets (3/22, 14%) were used most frequently to present immersive VR scenes. More detailed information about the VR systems is shown in Table 1.

**Table 1:** Degree of immersion, devices and construction approach for the virtual reality (VR) technology in the included studies.

Degree of immersion, study, and year			Device		Construction approach
**Nonimmersive VR**					
	Man et al [34], 2011	Computer		Geometry-based VR	
	Lancioni et al [38], 2015	Computer		Image-based VR	
	Hou et al [45], 2017	Computer and Kinect		Image-based VR	
	Klein et al [49], 2018	Display		Image-based VR	
	Tominari et al [24], 2021	Tablet		Image-based VR	
	Sun et al [47], 2020	Monitors, television, or projectors		Image-based VR	
	Tabafunda et al [43], 2020	Webcam and display		Image-based and geometry-based VR	
**Semi-immersive VR**					
	Siriaraya and Ang [48], 2014	Kinect sensor and projector		Geometry-based VR	
	Chapoulie et al [46], 2014	BARCO iSpace, glasses with advanced real-time tracking system, and ART wireless finger-tracking devices		Image-based VR	
	Benoit et al [41], 2015	BARCO iSpace, glasses with advanced real-time tracking system, and ART wireless finger-tracking devices		Image-based VR	
	Manera et al [42], 2016	A Barco OverView OLSF-721 full HD 3D stereoscopic LED^a^ video wall, and Volfoni Edge 1.2 active 3D LCD^b^ shutter glasses		Image-based VR	
**Immersive VR**					
	Tsao et al [44], 2019	VR case headset		Geometry-based VR	
	Xu and Wang [25], 2020	HTC VIVE headset		Geometry-based VR	
	Yu and Choi [18], 2018	VIVE and Oculus headsets		Image-based VR	
	Klein et al [49], 2018	Headset VR without detailed information		Image-based VR	
	Webber et al [35], 2020	Oculus Go headset		Image-based VR	
	Saredakis et al [26], 2020	Oculus Go headset		Image-based VR	
	Sun et al [47], 2020	Headset VR without detailed information		Image-based VR	
	Coelho et al [36], 2020	Samsung Gear VR with a smartphone and the Oculus Rift		Image-based VR	
	Yahara et al [32], 2021	Oculus Go headset		Image-based VR	
	Saredakis et al [33], 2021	Oculus Go headset		Image-based VR	
	Huang and Yang [37], 2022	VIVE headset		Image-based VR	
	Niki et al [39], 2021	Oculus Go headset		Image-based and geometry-based VR	
	Afifi et al [40], 2021	Oculus Go headset		Image-based and geometry-based VR	

^a^LED: light-emitting diode.

^b^LCD: liquid-crystal display.

Next, we classified the approaches used in the included studies to construct VR environments into geometry-based VR and image-based VR [44]. Specifically, 7 (32%) studies used geometry-based VR, which allows users to experience simulated objects and scenes in a virtual environment created using specialized 3D model-building software (eg, 3DS Max, Unity, and AutoCAD). Meanwhile, 18 (82%) studies used images or videos from the real world to establish image-based VR through image processing, image-based rendering, or video editing. More information about the VR technologies used in the 22 included studies can be found in Multimedia Appendix 1.

### Characteristics of VR-RT Interventions

The VR-RT stimulus materials were classified according to the content of the VR scenes as virtual travel for addresses, old locations, old items, and personally relevant pictures or videos. In the included studies, virtual travel for addresses usually allowed participants to view places of interest on Google Maps; old locations were used to indicate historical architectural and infrastructural features such as parks, homes, streets, or buildings; old items were products previously used by the participants, including foods, clothing, artifacts, magazines, posters, music, and other similar items that were once part of their daily lives; and personally relevant pictures or videos were photos and videos of the participants or their friends or family members. We further divided the stimulus materials into personalized and general stimulus materials. The personalized stimulus materials were tailored for the participants on the basis of their past experiences while the general stimulus materials were generic resources applicable to a broad population.

Four (18%) studies used virtual travel for addresses as stimulus materials [26,32,33,35]. Although these studies allowed the participants to view preferred addresses on Google Maps, no correlation to a specific time period (eg, the 1960s) was identified in the studies. Therefore, we considered virtual travel for addresses to be general stimulus materials. Eight (36%) and 6 (27%) studies used old locations [36,37,39-41,43,44,46] and old items [24,25,34,45,48,49], respectively, to evoke the participants’ memories. Most of the stimulus materials in these categories pertained to the period between the 1940s and the 1980s and specifically to the participants’ periods of childhood and adolescence, as shown in Table 2. Notably, 2 (9%) studies used personalized old locations based on the participants’ experiences [36,40], whereas the other studies (12/22, 55%) involving old items and old locations used general stimulus materials. Four (18%) studies used pictures or videos of the participants and their family members in the past as stimulus materials [18,38,40,42].

**Table 2:** The stimulus materials and intervention characteristics of virtual reality-based reminiscence therapies.

Stimulus materials and type		Content	Intervention duration, mean (SD)
**Virtual travel for addresses**			
	General	Google Maps street view [26,32,33,35].	23.75 (14.93) min for 3.50 (3.11) sessions, 2.00 (1.41) wks
	Personalized	—^a^	—^a^
**Old locations**			
	General	Historic houses between the 1950s and 1980s [37,39,44]; familiar and unfamiliar locations in the past [41,43,46].	9.44 (6.15) min for 6.75 (11.50) sessions, 3.75 (5.50) wks
	Personalized	Favorite locations or destinations from the past [40]; childhood houses and workplace locations as well as leisure and religious venues [36].	20.00 (14.14) min for 2.50 (2.12) sessions, 1.50 (0.71) wks
**Old items**			
	General	Items in a home setting [25,48] and a convenience store [34]; food, clothing, live, transportation, and festival items [45]; material and cultural artifacts corresponding to childhood [24]; old items from between the 1940s and 1970s [49].	24.00 (14.07) min for 4.20 (4.44) sessions, 3.00 (3.08) wks
	Personalized	—^a^	—^a^
**Personally relevant pictures or videos**			
	General	—^a^	—^a^
	Personalized	Family photos and videos [40]; personal old photos [18,38,42].	13.33 (14.43) min for 49.50 (68.59) sessions, 2.50 (2.60) wks

^a^Data not available.

We further summarized and calculated the mean intervention durations of the analyzed VR-RTs. After excluding 4 (18%) studies that did not report the intervention duration [18,43,44,48], the results showed that studies using general old items had the longest mean single-session duration (mean 24.00, SD 14.07 min), while studies using general old locations (6/22, 27%) had the shortest mean single-session duration (mean 9.44, SD 6.15 min). In addition, interventions using personally relevant pictures or videos had the most sessions according to the reported designs (mean 49.50, SD 68.59 sessions). However, except for 1 (5%) study that reassessed participants 3 to 6 months after the intervention [24], the remaining studies (21/22, 96%) conducted no follow-up measure.

Furthermore, 5 (23%) included studies compared the effectiveness of VR-RTs with traditional paper-based [24,25,33,41] and talk-based RTs [25,39]. In paper-based RTs, the stimulus materials are presented on paper; in talk-based RTs, participants’ memories are recalled through conversation. Three (14%) included studies compared the effectiveness of nonimmersive VR-RTs with nontreatment [33,41,46], semi-immersive VR-RT [41,46], or immersive VR-RT [33], and 2 (9%) of these studies also compared the effectiveness of using familiar versus unfamiliar stimulus materials in VR-RTs [41,46].

### Outcome Measures

#### Life Quality

As shown in Table 3, 4 (18%) studies [24,33,34,36] estimated the effects of VR-RTs on the quality of life of older adults via statistical tests, such as analysis of variance, the 2-tailed *t* test, and the Mann-Whitney *U* test. The participants’ quality of life was assessed in various domains, including psychometric status, ability to live independently, and physical health. One (25%) of these studies indicated that VR-RT using old items had significant beneficial effects on older adults’ psychometric status (*P*<.01) [24], while the remaining studies (3/22, 14%) [33,34,36] found no substantial differences in quality of life from before to after the intervention. Moreover, paper-based RT [24,34] or nontreatment [33] were not found to have substantial beneficial effects on quality of life.

**Table 3:** Effects of virtual reality–based reminiscence therapies (VR-RTs) on quality of life.

Outcome and measurement tools for older adults’ quality of life		Stimulus materials	VR-RTs	NVR-RTs^a^
	Lawton Instrumental Activities of Daily Living scale	Old items [34]	No effect	No effect
	Quality of life in Alzheimer disease	Virtual travel for addresses [33]	No effect	No effect
	Three-item loneliness scale	Virtual travel for addresses [33]	No effect	No effect
	Revised Philadelphia Geriatric Center Morale Scale	Old items [24]	Positive effect^b^ (*P*<.01)	No effect
	EUROHIS-QOL-8^c^ scores	Old locations [36]	No effect	None^d^

^a^NVR-RT: non–virtual reality–based reminiscence therapy.

^b^A significant difference between virtual reality–based reminiscence therapies and non–virtual reality–based reminiscence therapies.

^c^EUROHIS-QOL-8: European Health Interview Survey-Quality of Life 8-item index.

^d^No measurement for non–virtual reality–based reminiscence therapy.

#### Cognitive-Related Functions

As shown in Table 4, 13 (59%) studies evaluated the effects of VR-RTs on cognitive-related functions, including emotion, memory performance, and cognitive status [24-26,32-39,41,46]. Specifically, 5 (39%) of these studies assessed emotions (eg, anxiety, apathy, and depression) [24,32,33,37,39], 8 (62%) studies estimated memory performance [24-26,34,35,38,41,46], and 4 (31%) studies evaluated cognitive status [24,33,36,37]. As 1 (8%) study performed a qualitative data analysis [35], we reviewed and analyzed qualitative and quantitative results. Regarding quantitative data, 2 (17%) studies measured and reported the changes in outcome values from before to after the intervention [32,38], and the remaining 10 (83%) studies used statistical tests, such as the paired *t* test, Mann-Whitney *U* tests, and Wilcoxon signed rank test, to compare the outcome changes.

**Table 4:** Effects of virtual reality–based reminiscence therapies (VR-RTs) on cognitive-related functions.

Outcome and measurement tools		Stimulus materials	VR-RTs	NVR-RTs^a^
**Anxiety**				
	State-Trait Anxiety Inventory	Old locations [39]	Positive effect	None^b^
	State-Trait Anxiety inventory	Virtual travel for addresses [32]	Positive effect	None^b^
**Apathy**				
	Apathy Evaluation Scale	Virtual travel for addresses [32]	Unclear	None^b^
	Apathy Evaluation Scale	Virtual travel for addresses [33]	No effect	No effect
**Depression**				
	Geriatric Depression Scale	Virtual travel for addresses [33]	No effect	No effect
	Center for Epidemiological Studies Depression scale	Old locations [37]	Positive effect	None^b^
	Multidimensional Observation Scale for Elderly Subjects	Old items [24]	No effect	No effect
**Memory**				
	Verbal engagement	Personally relevant pictures or videos [38]	Positive effect	None^b^
	Multifactorial Memory Questionnaire	Old items [34]	Positive effect	Positive effect
	Fuld Object-Memory Evaluation	Old items [34]	Positive effect	Positive effect
	Performance in Autobiographical Fluency Task	Old locations [41]	Positive effect	No effect
	Past reconstruction	Virtual travel for addresses [35]	Positive effect	None^b^
	TEMPau scale	Old items [25]	Positive effect	Positive effect
	Word fluency test	Old items [24]	No effect	No effect
	Verbal fluency test	Virtual travel for addresses [26]	Positive effect	None^b^
	Verbal fluency test	Old locations [46]	Positive effect	No effect
**Cognitive status**				
	Clinical Dementia Rating	Old locations [37]	No effect	None^b^
	Mini-Mental State Examination	Old locations [37]	No effect	None^b^
	Mini-Mental State Examination	Old items [24]	Positive effect	Positive effect
	Cognitive Abilities Screening Instrument	Old locations [37]	No effect	None^b^
	Trail-making test parts A and B	Old items [24]	No effect	No effect
	Neuropsychiatric Inventory	Old locations [36]	No effect	None^b^
	Addenbrooke Cognitive Examination III	Virtual travel for addresses [33]	No effect	No effect

^a^NVR-RT: non–virtual reality–based reminiscence therapy.

^b^No measurement for non–virtual reality–based reminiscence therapy.

The effectiveness of VR-RTs for mitigating anxiety was verified in 2 studies that used old locations as stimulus materials [32,39]. However, inconsistent results were reported in terms of the effectiveness of VR-RTs in reducing apathy and depression. Three included studies assessed depression [24,33,37], and only 1 study found a positive effect of VR-RTs when using virtual travel for addresses [37]. The effect of VR-RTs on apathy was unclear due to a pair of opposing outcomes in 1 (50%) study [32].

Among the included studies that addressed memory, 7 (88%) out of 8 reported that VR-RTs improved participants’ memories [25,26,34,35,38,41,46]. All related (2/2, 100%) studies found that familiar old locations were substantially more effective than unfamiliar environments in VR-RTs [41,46]. In contrast, no substantial differences in effectiveness were observed between nonimmersive and semi-immersive VR-RTs. VR-RTs were found to have similar positive effects on memory as paper-based RT [25,34] and talk-based RT [25].

Only 1 (25%) included study revealed that VR-RTs could significantly improve the participants’ cognitive status (*P*<.01), assessed by the Mini-Mental State Examination after the intervention. At the same time, no substantial positive effect was found during follow-up measures [24]. The remaining 3 (75%) studies showed no substantial beneficial effect of VR-RTs on cognitive enhancement [33,36,37]. Similarly, a paper-based RT did not benefit cognitive status [33]. However, none of the included studies showed substantial declines in cognitive status after VR-RT use, suggesting that VR-RTs could be used to maintain the cognitive status of individuals with cognitive impairment.

#### User Satisfaction

A total of 14 (64%) of the 22 studies assessed user satisfaction with the VR-RTs [25,26,32,33,35,36,39-43,46,48,49], of which, 11 (79%) studies estimated the side effects [25,26,32,33,35,36,39-42,46], 7 (50%) studies evaluated user acceptability [26,32,35,40,42,43,49], 4 (28.6%) studies examined the immersiveness of VR [25,40,41,46], and 8 (57%) studies examined engagement with the intervention [25,26,33,36,40,41,46,48]. The results of user satisfaction are summarized in Table 5. One (7%) study used the changes in outcome values from before to after the intervention to estimate the side effects of VR-RTs, as well as user acceptability and engagement [32]; 5 (36%) studies assessed user satisfaction via qualitative data analysis [35,36,43,48,49]; and 9 (64%) studies evaluated user satisfaction via statistical analysis [25,26,33,36,39-42,46].

**Table 5:** User satisfaction with virtual reality–based reminiscence therapies (VR-RTs) and non–VR-based RTs (NVR-RTs).

Outcome and measurement tools		Stimulus materials	VR-RTs	NVR-RTs
**Side effects**				
	Simulator Sickness Questionnaire	Virtual travel for addresses [33]	No effect	None^a^
	Simulator Sickness Questionnaire	Virtual travel for addresses [26]; old locations [36]	Positive effect	None^a^
	Self-report numerical rating scale	Old locations [40]; old items [25]; personally relevant pictures or videos [40,42]	No effect	No effect
	Self-report numerical rating scale	Old locations [41,46]	No effect	None^a^
	Self-report numerical rating scale	Virtual travel for addresses [32]; old locations [39]	No effect	None^a^
	Interview	Virtual travel for addresses [35]	No effect	None^a^
**User acceptability**				
	Self-report numerical rating scale	Virtual travel for addresses [26,32]	Positive effect	None^a^
	Self-report numerical rating scale	Personally relevant pictures or videos [42]	Positive effect^b^ (*P*=.04)	Positive effect
	Self-report numerical rating scale	Old locations [40]; personally relevant pictures or videos [40]	Positive effect	Positive effect
	Interview	Old locations [43]	Positive effect	None^a^
	Interview	Virtual travel for addresses [35]	NE^c^	None^a^
	Observation	Old items [49]	Positive effect	None^a^
**Immersion**				
	Self-report numerical rating scale	Old locations [41,46]; old items [25]	Positive effect	None^a^
	Self-report numerical rating scale	Old locations [40]; personally relevant pictures or videos [40]	Positive effect	Positive effect
**Engagement**				
	Human and automated coding	Personally relevant pictures or videos [40]; old locations [40]	Positive effect^b^ (*P*=.004)	Positive effect
	Self-report numerical rating scale	Virtual travel for addresses [26]; old locations [41,46]	Positive effect	None^a^
	Self-report numerical rating scale	Virtual travel for addresses [33]; old items [25]	Positive effect	Positive effect
	Observation	Old locations [36]; old items [48]	Positive effect	None^a^

^a^No measurement for non–virtual reality–based reminiscence therapies.

^b^A significant difference between virtual reality–based reminiscence therapies and non–virtual reality–based reminiscence therapies.

^c^NE: negative effect.

The results of 9 (64%) studies suggested that the VR-RTs were highly safe, with negligible side effects [25,32,33,35,39-42,46]. In contrast, 2 (14%) studies found that VR-RTs using virtual travel for addresses or old locations triggered moderate or high side effects in participants [26,36]. Specifically, discomfort from wearable devices, vertigo, a sensation of falling, eye strain, nausea, fullness in the head, burping, and blurred vision were reported [26,36]. No substantial differences in side effects were identified between VR-RTs and paper-based RT [25,42] or talk-based RT [25,40].

In 6 (86%) studies, participants with cognitive impairment reported that the VR-RTs were enjoyable, attractive, easy to use, acceptable, and highly satisfying [26,32,40,42,43,49]. However, a few participants in 1 (14%) study claimed that the VR-RTs had little value and preferred to avoid virtual travel for addresses due to sadness [35]. In addition, the users reported significantly higher satisfaction levels with VR-RTs using personally relevant pictures or videos than with a paper-based RT (*P*=.04) [42]. The VR-RTs were also highly immersive and engaging [25,26,33,36,40,41,46,48], with some studies reporting higher engagement levels than those recorded for talk-based RT [40].

#### Risk of Bias

**Figure 2:**
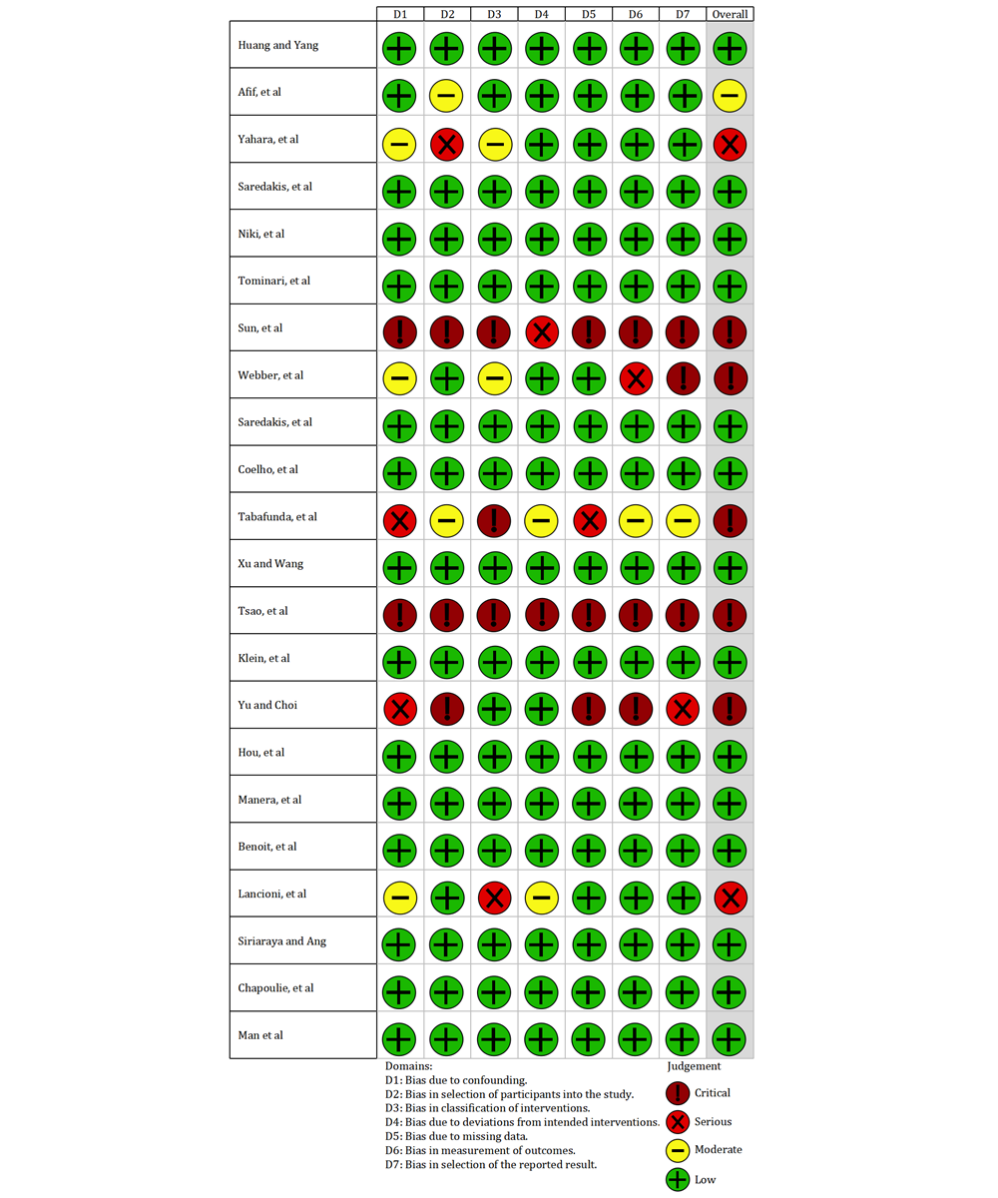
Assessment results of the risk of bias.

Of the 22 included trials, 14 (64%) articles had a low risk of bias, 1 (5%) had a moderate risk of bias, 2 (9%) had a serious risk of bias, and 5 (23%) had a critical risk of bias (Figure 2 [18,24-26,32-49]).

## Discussion

### Principal Findings

In this systematic review, 22 studies were synthesized comprehensively to evaluate the effects of VR-RTs on older adults with cognitive impairment. The results revealed that VR-RTs could effectively improve emotion and memory but had limited potential to improve quality of life and cognitive status. Concerning user satisfaction, although 2 (9%) studies reported some side effects and 1 (5%) study reported that VR-RTs could trigger negative emotions, most participants reported negligible side effects, indicating that the VR-RTs were safe and enjoyable, as indicated by high levels of immersion and engagement. We further discuss the specific impacts and implications of VR-RTs below.

### Effect of VR-Based RTs on Quality of Life

For older adults, RT can enhance life satisfaction and self-confidence by transferring previously gained social skills and enjoyable experiences to real life [50,51]. RTs also allow individuals to reassess and resolve past conflicts, allowing people to gain meaning and a sense of belonging in daily life [52]. However, in contrast with previous studies [51,53,54], we found that most of the studies included in our review showed no substantial positive effect of VR-RTs or traditional RTs on older adults’ quality of life, except for 1 (5%) RCT in which VR-RTs effectively increased participants’ Philadelphia Geriatric Center Morale Scale scores [24]. The inconsistent effects of VR-RTs and RTs on the participants’ quality of life may be attributable to differences in the intervention modalities and settings [55]. For example, one previous study reported that RT was substantially effective only if delivered in 6 or 7 sessions [53]. In addition, most included studies assessed VR-RTs in non-RCTs, possibly leading to result bias. We recommend that additional RCTs with longer intervention durations be conducted to investigate the effect of VR-RTs on quality of life.

### Effect of VR-Based RTs on Cognitive-Related Outcomes

We analyzed and synthesized the effects of VR-RTs on older adults’ emotions, memory, and cognitive status. According to our results, VR-RTs effectively improved anxiety and memory, consistent with the findings of previous studies on VR-based or RT interventions [54,56]. VR technologies using diverse sensory stimuli considerably enhanced the participants’ motivation and interest in the training and elicited pleasant feelings [57]. Therefore, VR technology undeniably played a critical role in RT interventions intended to decrease anxiety and improve emotions. Furthermore, most of the included studies [25,26,41,46] verified the effectiveness of VR-RTs for enhancing autobiographical memory, which pertains to a person’s recollection of specific and personal events from their past experiences [58]. This finding is consistent with those of a previous study, suggesting that exposure to an immersive environment containing numerous concrete cues from a person’s youth can improve their autobiographical recollection [59]. RT uses memory triggers to recall the long-term memories of people with cognitive impairment [60]. Accordingly, RTs combined with immersive environments were shown to evoke substantial improvements in autobiographical memory. In particular, VR-RTs using familiar old locations had stronger positive effects on autobiographical memory than VR-RTs using unfamiliar environments [41,46]. In addition, 1 (5%) included study revealed that VR-RTs substantially enhanced the temporal orientation of older adults with MCI [24]. In another study, VR-RTs considerably improved participants’ objective memory performance, especially their recall of episodic memories immediately and after a delay [34]. Therefore, the potential applications of VR-RTs for memory improvement in older adults with cognitive impairment are enormous.

However, most of the included studies reported that VR-RTs had no substantial effects on depression and apathy. The potential reasons for this lack of effect might be the intervention designs and participants. For example, group RT, which allows several patients to be treated together, was verified to yield greater positive effects and to be more cost-effective than RT with only 1 patient [61]. In addition, RT is not considered an appropriate means of reducing depression in people with severe dementia due to the strong relationship between depression and cognitive impairment [51]. Therefore, we suggest that the VR-RTs may have been somewhat effective for older adults with MCI, given that beneficial interactions in VR-RTs can allow participants to revisit past problems and achieve goals [62]. Although previous studies found that RT had a substantial positive effect on apathy [54,60], the impacts of VR-RTs on apathy and the underlying mechanisms remain unclear due to the limited number of studies. In future studies, it will be crucial to investigate the effects of VR-RTs on depression and apathy in older adults with specific cognitive impairments.

Furthermore, only 1 (25%) study [24] reported that VR-RTs significantly (*P*<.01) improved participants’ cognitive status among the included 4 (100%) studies [24,25,33,41]. Previous studies have reported inconsistent effects of RT on cognitive status. Some studies identified a significant moderate or small effect size of RT on cognitive status [51,55]. In contrast, other studies reported no statistically significant effects of RT [54,63]. This discrepancy may be attributable to the various sensitivities and specificities of the assessment tools used to measure cognitive status [59]. Differences in the quality and methodology of studies may also have affected the consistency of the results [64]. Although no significant effectiveness of VR-RTs was reported in improving the participants’ cognitive status, the interventions were beneficial in maintaining cognitive status and preventing functional degradation [10], consistent with the findings of previous studies [54,63]. People with cognitive impairment, especially dementia, experience progressive brain shrinkage, which exacerbates cognitive impairment [63]. It is crucial to conduct interventions to preserve cognitive status in this situation. Individual-based or care home-based interventions were verified to improve cognitive status more than general interventions [55]. Thus, we recommend the use of personalized VR-RTs as a nonpharmacological treatment to maintain the cognitive function of people with cognitive impairment.

### User Satisfaction With VR-Based RTs

Although VR-based interventions are commonly considered interesting and enjoyable, users may experience side effects due to perceptual discrepancies between the real and virtual environments [65]. Thus, the occurrence and severity of side effects must be considered when assessing user satisfaction with VR-RTs. Two (14%) included studies that used image-based VR reported short-duration side effects such as discomfort caused by wearable devices, eye strain, vertigo, nausea, spatial disorientation, and mild negative emotions [26,36]. Inappropriate placement and inadequate camera resolution were suggested as potential factors that may induce physiological discomfort during VR-RTs [26,36]. Participants also may experience spatial disorientation and confusion due to external verbal interference from reminiscence therapists [36]. Despite these potential side effects of VR-RTs, participants commonly expressed positive attitudes regarding the acceptability of VR-RTs. They were satisfied with the VR-RTs and considered them to be potentially beneficial.

Meanwhile, people preferred immersive VR-RTs to traditional RT or nonimmersive VR-RTs (eg, paper-based and flat screen–based RTs) [33,42]. The finding that immersive VR with multisensory stimulation can enhance biofeedback and presence in the virtual environment [66] prompted the implementation of VR-RTs. However, 1 (5%) included study found that the participants tended to avoid VR-RTs because of negative memories [35]. Hence, research scholars must adequately consider older adults’ experiences and avoid potentially adverse RT contexts [58].

In the included studies, the VR-RTs were commonly considered highly immersive and engaging. A high level of immersion gives older adults a sense of being physically together with their family members, especially during VR-RTs that allow older adults and their family members to interact [67]. Older adults who engaged more in the VR-RTs achieved a greater social presence, which prompted them to maintain secure attachments to their families [40]. Notably, older adults with MCI were found to engage more deeply in the VR-RTs than those with dementia, whereas the opposite pattern was observed for immersion [40]. The severity of cognitive impairment may limit physical mobility and interaction [68].

### Practical Implications

The included studies used a wide variety of VR technologies and RT content. Given the limited evidence regarding the effects of specific VR-RT programs, we provide preliminary insights into the design of the VR-RT interventions for older adults with cognitive impairment, including the immersion levels and construction approaches of VR technology, the stimulus materials, and the duration of the VR-RT intervention. We also discuss several challenges related to the implementation of VR-RTs.

### VR Technology

The immersion level can influence the effectiveness of VR-RTs and user satisfaction by affecting users’ sense of presence and engagement [69]. Although immersive VR provides high levels of immersion and engagement, it may cause older adults with severe dementia to experience an increased cognitive load [70]. This may occur because the allocation of brain and sensory resources increases with the degree of immersion in VR [71]. Among the studies included in our review, 2 (14%) studies using immersive VR-RTs reported that participants experienced several side effects [26,36]. In contrast, studies that used semi-immersive VR-RTs [41,42,46] or nonimmersive VR-RTs [72] reported no side effects. However, the optimal immersion level for effective VR-RTs remains uncertain due to the limited number of relevant RCTs. Given the potential side effects, we recommend increased caution when applying immersive VR to older adults.

Image-based VR was considered more acceptable by users compared with geometry-based VR, as it generated more positive emotions [39]. However, the included studies suggested that image-based VR is more likely to induce side effects than geometry-based VR [26,36]. Image-based VR uses highly realistic scenes [72], and the delay between the image presentation and the user’s motions might contribute to increased side effects [73]. Thus, we recommend that future studies on the design of image-based VR focus on reducing its side effects.

### VR-RT Interventions

The included studies used 4 types of stimulus materials associated with the earlier periods of the participants’ lives, consistent with the finding that individuals with Alzheimer disease exhibited better recall of memories from their youth than of recent memories [25]. Familiar stimulus materials were verified as superior to unfamiliar ones for improving the participants’ emotions and memory recall [33,41,43,46]. Thus, for VR-RTs, personalized stimulus materials related to the individual’s personal history are recommended [49]. In addition, although most of the included studies used visual materials as stimuli, aural materials are also suitable for VR-RTs as they confer benefits such as enjoyment and information encoding [34,43,74]. Therefore, personalized visual and aural stimulus materials associated with the participants’ younger days tend to be more effective than general stimulus materials in interventions for cognitive impairment; such materials may enhance cognitive abilities, emotional well-being, and quality of life.

The duration of a VR-RT intervention can also influence its effectiveness. A session duration of around 40 minutes and a total intervention duration of 8 to 12 weeks were considered the most beneficial for participants [64,65]. Although 72% of the users in 1 study left the VR environment after 36 minutes due to cybersickness [72], most included studies restricted the participants’ VR exposure time to <30 minutes. Moreover, most studies have only preliminarily evaluated VR-RTs through feasibility studies; thus, the optimal duration of a VR-RT has not been defined. More RCT studies are warranted to determine an appropriate duration of VR-RTs that elicits more positive effects with fewer side effects.

Despite the potential benefits of VR-RTs for older adults with cognitive impairment, the included studies also reported diverse concerns. For example, participants with cognitive impairment in one study expressed concerns regarding vision difficulties in the VR environment [43]. Another study suggested the importance of passive interactivity between patients and caregivers [43], while a third study suggested that an external disturbance could disrupt a participant’s attention [36]. VR-RTs may also induce negative emotions in participants due to the recall of unpleasant memories [35]. More attention should be given to the above-mentioned problems and the careful design of interventions to promote the implementation of VR-RTs for older adults with cognitive impairment.

### Limitations

This systematic review has several limitations. First, although the findings suggest that VR-RTs are potentially beneficial in improving emotions and memory functions, the methodological restrictions of the included studies prevented us from obtaining statistical evidence of these effects. A meta-analysis is recommended to determine the effectiveness of VR-RTs in terms of specific functional improvements in older adults with cognitive impairment. Second, as few of the included studies were RCTs comparing the effects of VR-RTs and traditional RTs on cognitive-related outcomes, it remains unclear whether VR-RTs differ significantly from traditional RTs regarding effectiveness. We recommend that future studies compare VR-RTs with traditional RTs through RCTs with large sample sizes and rigorous methodologies. The results of such studies may deepen our knowledge of the potential benefits and limitations of VR-RTs for older adults with cognitive impairment. Third, the intervention design and the participants’ demographic characteristics, including age, emotional status, and the severity of cognitive impairment, may influence the effectiveness of a VR-RT. However, we have limited insight into these factors’ potential influences and specific mechanisms. Future studies should also diversify the participant groups and VR-RT contents when designing treatment protocols.

### Conclusions

We performed this systematic review to provide insights into the effects of VR-RTs among older adults with cognitive impairment. The results reveal that VR-RTs have negligible side effects and high levels of engagement and thus can achieve high levels of user satisfaction. VR-RTs were shown to effectively improve memory functions and emotions and maintain the cognitive status of older adults with cognitive impairment. However, there is limited evidence to support the potential benefits of VR-RTs in terms of improving older adults’ quality of life. Moreover, personalized stimulus materials of VR-RTs were found to outperform general stimulus materials for enhancing or maintaining cognitive-related functions. In particular, multisensory stimulus materials related to experiences from their days of youth can considerably enhance the benefits of VR-RTs among older adults. However, some side effects of VR technology use, such as cybersickness and vision difficulties, have raised concerns. In summary, VR-RT applications appear to be a promising option for the nonpharmacological treatment of cognitive impairment.

## Appendix

Multimedia Appendix 1 Characteristics of the 22 papers included in the systematic review.

Multimedia Appendix 2 PRISMA (Preferred Reporting Items for Systematic Reviews and Meta-Analyses) checklist.

## Abbreviations

MCI: mild cognitive impairment
RCT: randomized controlled trial
RT: reminiscence therapy
VR: virtual reality
VR-RT: virtual reality–based reminiscence therapy

## Data Availability

The datasets generated and analyzed during this study are available from the corresponding author on reasonable request.
