# Supplementary material for: The Effects of Virtual Reality–Based Reminiscence Therapies for Older Adults With Cognitive Impairment: Systematic Review
Source: J Med Internet Res. 2024 Nov 12;26:e53348. doi: 10.2196/53348 (PMC11599890; doi:10.2196/53348)
Supplement: Multimedia Appendix 1 [file jmir_v26i1e53348_app1.docx]

**Appendix 1.** Characteristics of the 22 papers in the systematic review

| **Article** | **Study design** | **Subject characteristics** | **Health status** | **VR technology** | **Contents of the VR-RT** | **Contents of the non-VR intervention** | **Intervention** | **Measurement time points** | **Outcomes** |
| --- | --- | --- | --- | --- | --- | --- | --- | --- | --- |
| Huang, et al, 2022 Taiwan [36] | Pilot study | N=20 (female = 11);  Age (79.0±7.8) | Dementia | Device: VIVE Pro VR head-mounted display (HMD) (immersive VR);  Amount: 1;  Placement: head;  Form: sit on the chair;  Construction approach: Image-based VR | Using the controllers to turn on the radio to play the music and to look through the photo album and browse the photographs with a voice narrating the past meaningful situation and events about the photographs. They could also use the controller to hold rice to feed chickens. | None | 10~12 minutes/ session, 2 sessions per week, for 3 months | Pre and post; 3-6 months after | Zarit caregiver burden interview;  Cognitive abilities screening instrument;  Mini-mental state examination;  Clinical dementia rating;  Center for epidemiological studies depression |
| Afif, et al, 2021, USA [39] | Feasibility study | N=21 (female=18);  Age (83.10±9.76) | MCI or mild-to-moderate dementia | Device: Oculus Go (immersive VR);  Amount: 1;  Placement: head;  Form: sit on the chair;  Construction approach: Geometry-based VR and Image-based VR | Virtual adventures: Resident and family member chose five travel adventures among 25 possible preprogrammed adventures; Virtual travel life story: Resident and family member were taken back to favorite addresses or destinations from the past; Virtual family photos and videos: Resident and family member viewed their photos and video in a virtual family room, seated beside each other on a virtual couch. | phone call | 30 minutes/ session, 1 session/ per week, for 3 weeks | After each session | Side effect and satisfaction;  Immersion and engagement |
| Yahara, et al, 2021, Japan [31] | A case study | N=2 (female=1);  Age (86.00±8.49) | MCI | Device: Oculus Go (immersive VR);  Amount: 2;  Placement: head;  Form: not given, maybe sit on the chair;  Construction approach: Image-based VR | Implementation of Face-to-Face iVR Reminiscence  Session（describe their memorable places, and then used an app that can view Google map street view）; Implementation of Remote iVR Reminiscence Session（describe their memorable places, and then used an app that can view Google map street view）. | None | 10 minutes/session, 6 sessions for P1 and 8 sessions for P2 | Before and after each session | Burden of care;  State-trait anxiety inventory;  Apathy scale;  Side effect and satisfaction;  Attitude |
| Saredakis, et al, 2021 Australia [32] | Pilot study | N=43; Immersive VR: n=15 (female = 10);  Age (81.7±6.6);  Non-immersive VR: n=14 (female = 9);  Age (85.9±8.1);  Passive control: n=14 (female = 9);  Age (87±8.7) | Not given | Device: Oculus HMD (immersive VR) and a laptop computer (non-immersive VR);  Amount: 1;  Placement: head;  Form: sit on the chair;  Construction approach: Image-based VR | Viewed the reminiscence content by VR (Google Street View with Wander VR, according to the participants' experiences) and a laptop computer. | usual care | 20 minutes/session, 3 sessions in 2 weeks | Pre and post | Quality of life in alzheimer disease  three-item loneliness scale;  Apathy evaluation scale;  Addenbrooke cognitive examination;  Geriatric depression scale;  Simulator sickness questionnaire;  Session record |
| Niki, et al, 2021 Japan [38] | Pilot study | N=10 (female = 6);  Age (87.1±4.2) | Not given | Device: Oculus Go (immersive VR);  Amount: 1;  Placement: head;  Form: sit on the chair;  Construction approach: Image-based VR and Geometry-based VR | Live-action images of Itsuka Kita Michi in Miroku no Sato, a theme park in Fukuyama City, Hiroshima Prefecture; Computer graphics images of Japanese town model set vol. 3 from Unity asset store and edited the CG images using Unity ( arcades, cafeterias, sunken hearths, dagashi shops (old Japanese candy stores), downtown, and fields). | None | 10 minutes / session for two sessions | After each session | State-trait anxiety inventory;  The safety of the reminiscence and the preference |
| Tominari, et al, 2021 Japan [24] | Randomized controlled trial | N=52;  VR: N=26 (female = 19);  Age (85.1) Non-VR: n=26 (female = 21);  Age (87.0) | MCI | Device: tablet iPads (non-immersive VR);  Amount: 1;  Placement: infront;  Form: Not given (maybe sit);  Construction approach: Image-based VR | Viewed 360-degree panoramic displays that could be enlarged or reduced freely on tablets (Photos of material and cultural artifacts corresponding to the childhoods of participants: plays, school classrooms, a house with a hearth or fireplace, holiday doll decorations, a shopping street, candy shops, interiors of cafeterias, and an appliance store) | Viewed four printed panoramic covering 90 degrees each | 30~45 minutes / session, 1 session per week, for 8 weeks | Pre and post | Revised Philadelphia Geriatric Center Morale Scale;  Mini-mental state examination;  Multidimensional observation scale for elderly subjects;  Trail making test parts a and b;  Word fluency test |
| Sun, et al, 2020 Canada [46] | Prototype | None | None | Device: 1) immersive VR with head mounted displays (immersive VR); 2) monitors, television, or projectors to visualize and engage with the content (non-immersive VR);  Amount: not given;  Placement: head/infront;  Form: sit, stand, room scale;  Construction approach: Image-based VR | None | None | None | Pre and post | Frequency of responsive incident, behaviours and medication use;  Facial and eye tracking |
| Webber, et al, 2020 Australia [34] | Pilot study | N=7 (none);  Age (none); | Not given | Device: Oculus Go VR system (immersive VR) Tablet computer (non-immersive VR);  Amount: 1;  Placement: head;  Form: Not given;  Construction approach: Image-based VR | Google Maps Street View with Wander VR, according to the participants' experiences and Google Maps with Tablet computer (Apple iPad), according to the participants' experiences. | None | 45 minutes/session for two sessions | After each session | Forms of reminiscence prompted by virtual visits;  Comparing device types for digital reminiscence;  Limitations of digital mapping technologies for reminiscence;  Limitations of device usability for older adults and facilitators;  Perceived value of virtual visits |
| Saredakis, et al, 2020 Australia [26] | Feasibility study | N=17 (female = 10);  Age (87.3±6.3) | Not given | Device: Oculus Go HMD (immersive VR);  Amount: 1;  Placement: head;  Form: sit on the chair;  Construction approach: Image-based VR | Google Street View with Wander VR, according to the participants' experiences. | None | 20 minutes/session for 2 sessions | Pre and post | Phonemic verbal fluency;  Semantic verbal fluency;  Simulator sickness questionnaire total;  Simulator sickness questionnaire nausea;  Simulator sickness questionnaire oculomotor;  Simulator sickness questionnaire disorientation;  Expectations/enjoyment;  Debriefing questionnaire |
| Coelho, et al, 2020 Portugal [35] | Pilot study | N=9 (female = 6);  Age (85.6±7.4) | Dementia | Device: Samsung Gear VR with a Samsung S7 smartphone and the Oculus Rift (immersive VR);  Amount: 1;  Placement: head;  Form: sit on the chair;  Construction approach: Image-based VR | Individuals with dementia were exposed to four reminiscence videos (childhood houses and workplace locations, as well as leisure and religious venues) | None | 10 minutes/session, for 4 sessions in two weeks | Pre and post | Neuropsychiatric inventory;  Participants’ engagement and behavior;  Simulator sickness, psychological and behavioral symptoms of dementia during reminiscence sessions |
| Tabafunda, et al, 2020 Canada [42] | Feasibility study | N=3 (none) | Dementia | Device: webcam-based head-tracking and display Looking Glass (a holographic display presenting horizontal stereoscopic depth) (non-immersive VR);  Amount: 1 for each;  Placement: infront;  Form: not given;  Construction approach: Image-based VR and Geometry-based VR | Videos of the proposed systems with familiar environment were shown to patients and their caregivers for feedba  ck. | None | Not given | None | Interest;  Perception |
| Xu and Wang, 2020 China [25] | Feasibility study | N=30  VR: n=10 (female = 5);  Age (76.7±5.5);  Photo: n=10 (female = 8);  Age (79.4±2.0);  Blank: N=10 (female = 7);  Age (78.5±3.4) | Alzheimer’s disease | Device: HTC Vive Focus headset (immersive VR);  Amount: 1;  Placement: head;  Form: sit;  Construction approach: Geometry-based VR | Providing stimuli to each subject and asked them to view for 2 min freely (virtual reminiscence room resembled a Chinese rural cottage in the 1970s with an area of 30 square meters. In the room, there were classic old objects from the past). | Photo: providing subjects with 20 color print photos in which the reminiscence objects were identical to those in the virtual reminiscence room;  Blank : only talk without visual stimuli provided to subjects. | 2 minutes/ session for two sessions | Post | TEMPau scale; Verbal outputs;  Questionnaires for motivation, presence, sickness |
| Tsao, et al, 2019, Taiwan [43] | Prototype | None | None | Device: VR headset (immersive VR) /smartphone(AR);  Amount: 1;  Placement: head/hand;  Form: sit on the chair/stand;  Construction approach: Geometry-based VR | The AR was used to be familiar with and choose the tours and the VR was then used to experience the old music, picture and historic house. | None | None | None | None |
| Klein, et al, 2018 Germany [48] | Prototype | N=55  Jukebox: n=24 (female = 19);  Age (82.63±1.71); Pyramid: n=25 (female = 20);  Age (82.32±1.27);  Binoculars: n=6 (female = 3);  Age (74.67±5.71) | Dementia | Device: Jukebox-Monitor (non-immersive VR); Pyramid-Monitor (non-immersive VR); Binoculars-smart phone +VR (immersive VR);  Amount: 1 for each;  Placement: infront;  Form: sit or lie;  Construction approach: Image-based VR | Jukebox: combines music from the past with relevant images to target different senses and stimulate memories; Pyramid: combines image material from the most formative and influential spans of time from the lives of participants, with thematically relevant sounds; Binoculars: The “Binoculars” function as a window into the past. The interaction with the zooming wheel allows the user to move from more recent content (mostly material from the 1970s and 1960s) to more ancient content (mostly material from the 1950s and late 1940s) in a virtual-reality environment. | None | 12~20 minutes | Post | Usability assessment;  Categorial system |
| Yu, et al, 2018 Korea [18] | Prototype | None | None | Device: VIVE and Oculus (immersive VR);  Amount: 2 products;  Placement: head;  Form: maybe sit on the chair;  Construction approach: Image-based VR | Identifying the photo-based contents of the dementia patients (three tasks with increasing difficulty). | None | None | None | None |
| Hou, et al, 2017 Taiwan [44] | Prototype | N=59 (female = 40);  Age (63.4±7.9) | Not given | Device: Windows Presentation Foundation and Kinect (non-immersive VR);  Amount: 1;  Placement: infront;  Form: sit on the chair;  Construction approach: Image-based VR | Tasks about selecting suitable objects from 25 familiar objects based on the participants' experiences. | None | 30 minutes | None | Scores for the game;  Montreal cognitive assessment and simple mental / cognitive status scale;  Mini mental status examination |
| Manera, et al, 2016 France [41] | Feasibility study | MCI: n=28 (female = 13);  Age (75.0±6.8);  Dementia: n=29 (female = 12);  Age (76.3±7.2) | MCI or dementia | Device: a Barco OverView OLSF-721 full HD 3D stereoscopic LED video wall, Volfoni Edge 1.2 active 3D LCD shutter glasses, wireless mouse (semi-immersive VR);  Amount: 1;  Placement: infront;  Form: site on the chair;  Construction approach: Image-based VR | Using the mouse to move a grey rectangle shape over the target character, and to click a mouse button to select the character in the virtual environment. | Selecting the characters by placing green rectangle shapes over the 2D version of the VR scenes printed on A3 paper sheets | 5 minutes | Post | Acceptability of the intervention |
| Benoit, et al, 2015, France [40] | Feasibility study | N=18 (female = 7);  Age (68.2±7.8) | Healthy | Device: display (non-immersive VR) and VR hardware (BARCO iSpace, glasses with Advanced Realtime Tracking system, and ART wireless finger-tracking devices) （semi-immersive VR）;  Amount: 1;  Placement: infront;  Form: sit on the chair;  Construction approach: Image-based VR | One condition using display to show the familiar photo and two conditions using semi-immersive VR to present the familiar photo and unfamiliar photo. | Gray screen | A maximum of 15 minutes | After each session | Total number of response;  Remember;  Anxiety;  Emotion;  Motivation;  Security;  Fatigue;  Familiarity;  Cybersickness;  Presence |
| Lancioni, et al, 2015 Germany [37] | Pilot study | N=16 (female = 11);  Age (82.63±6.74) | Moderate Alzheimer’s disease | Device: a computer system with screen and sound amplifier, a microswitch, and specific software (non-immersive VR);  Amount: 1;  Placement: infront;  Form: sit on the chair;  Construction approach: Image-based VR | The first program was shown video-recorded sequences of a virtual partner (i.e., a woman matching typical caregiving figures) who greeted him or her, presented questions (relevant topics) for engagement/reminiscence, and provided positive attention, and guidance (i.e., prompts/encouragements); The second program version replaced the appearance of the virtual partner and the questions with photos or 2 to 4s video clips of the participant him- or herself in special circumstances (e.g., children’s weddings or other celebrations) or of relevant people/events and places. | None | 5 minutes/ session, 2~3 sessions per day, for 73~122 sessions | Pre and post | Intervals with verbal Engagement/reminiscence;  Microswitch activations |
| Siriaraya, et al, 2014 England [47] | Prototype | N=20 | Dementia | Device: Kinect sensor, projector (semi-immersive VR);  Amount: 1;  Placement: infront;  Form: sit;  Construction approach: Geometry-based VR | Virtual world prototypes: An avatar placed in a virtual room with objects from the past (old posters, magazines, TVs, books). A radio was also included in the virtual room playing music of that period. prticipants can pick up items in the room; Virtual tour prototype: the participant was taken through a virtual river trip; Gardening Prototype: the participant would work together with the caregivers to design a virtual garden. | None | None | Post | Suggestions; Usability assessment |
| Chapoulie, et al, 2014 France [45] | Prototype | N=13 (female = 5);  Age (66.84±4.33) | Healthy | Device: display (non-immersive VR) and VR hardware (BARCO iSpace, glasses with Advanced Realtime Tracking system, and ART wireless finger-tracking devices) (VR);  Amount: 1;  Placement: infront;  Form: sit on the chair;  Construction approach: Image-based VR | Participants were exposed into familiar photo presented by display, familiar image-based semi-immersive environment, and unfamiliar image-based semi-immersive environment and allowed to navigate using the pointing gesture in image-based semi-immersive environment. | Gray screen | 45 seconds | After each session | Remember responses (verbal fluency test);  Familiarity question;  General questionnaire (emotion, motivation, and security);  Presence questionnaire (general presence and spatial presence, experienced realism and involvement, sickness, fatigue or anxiety during experiment) |
| David, et al, 2011 Hong Kong [33] | Pilot study | N=44  VR: N=20 (female = 17);  Age (80.30±1.21);  Non-VR: N=24 (female = 22);  Age (80.28±1.31) | Dementia | Device: computer (non-immersive VR);  Amount: 1;  Placement: infront;  Form: sit on the chair and use the joystick or the keyboard;  Construction approach: Geometry-based VR | Scenarios: a home setting and a convenience shop; Gradation structure: (tasks ranged from simple to more complex); Tasks: find and pick the correct items after a period of distraction. | paper‐and‐pencil tasks | 30 minutes/session, 2~3 sessions/ per week, for 10 sessions | Pre and post | Hong kong Chinese version of the lawton instrumental activities of daily living scale;  Fuld object memory evaluation-total encoding;  Fuld object memory evaluation-total retrieval;  Fuld object memory evaluation-delayed recall;  Multifactorial memory questionnaire-contentment;  Multifactorial memory questionnaire-ability;  Multifactorial memory questionnaire-strategy |
